# High expression of GMNN predicts malignant progression and poor prognosis in ACC

**DOI:** 10.1186/s40001-022-00950-2

**Published:** 2022-12-20

**Authors:** Xinzhao Zhao, Xuezhou Zhang, Shixiu Shao, Qingbo Yang, Chengquan Shen, Xuecheng Yang, Wei Jiao, Jing Liu, Yonghua Wang

**Affiliations:** 1 Department of Urology, The Affiliated Hospital of Qingdao University, Qingdao, Shandong China; 2 Department of Research Management and International Cooperation, The Affiliated Hospital of Qingdao University, Qingdao, Shandong China

**Keywords:** Adrenocortical carcinoma, GMNN, Signature, Prognosis, TCGA

## Abstract

**Background:**

Adrenocortical carcinoma (ACC) is a rare endocrine neoplasm, which is characterized by poor prognosis and high recurrence rate. Novel and reliable prognostic and metastatic biomarkers are lacking for ACC patients. This study aims at screening potential prognostic biomarkers and therapeutic targets of ACC through bioinformatic methods and immunohistochemical (IHC) analysis.

**Methods:**

In the present study, by using the Gene Expression Omnibus (GEO) database we identified differentially expressed genes (DEGs) in ACC and validated these DEGs in The Cancer Genome Atlas (TCGA) ACC cohort. A DEGs-based signature was additionally constructed and we assessed its prognosis and prescient worth for ACC by survival analysis and nomogram. Immunohistochemistry (IHC) was used to verify the relationship between hub gene–GMNN expressions and clinicopathologic outcomes in ACC patients.

**Results:**

A total of 24 DEGs correlated with the prognosis of ACC were screened from the TCGA and GEO databases. Five DEGs were subsequently selected in a signature which was closely related to the survival rates of ACC patients and GMNN was identified as the core gene in this signature. Univariate and multivariate Cox regression showed that the GMNN was an independent prognostic factor for ACC patients (*P* < 0.05). Meanwhile, GMNN was closely related to the OS and PFI of ACC patients treated with mitotane (*P* < 0.001). IHC confirmed that GMNN protein was overexpressed in ACC tissues compared with normal adrenal tissues and significantly correlated with stage (*P* = 0.011), metastasis (*P* = 0.028) and Ki-67 index (*P* = 0.014).

**Conclusions:**

GMNN is a novel tumor marker for predicting the malignant progression, metastasis and prognosis of ACC, and may be a potential therapeutic target for ACC.

**Supplementary Information:**

The online version contains supplementary material available at 10.1186/s40001-022-00950-2.

## Introduction

Adrenocortical carcinoma (ACC) is an orphan endocrine illness with an occurrence of between 0.5 and 2 for each million populace each year [1]. The majority of ACC patients had metastasis at the time of their initial diagnosis due to the challenges in early diagnosis, which leaves them with few options for treatment and very poor prognosis [2]. Patients with localized and regionalized ACC have a potential for cure with complete surgical resection, but a mass of patients still develop recurrent or metastatic disease [3, 4]. Although mitotane is approved in the treatment of metastatic ACC, the therapeutic effectiveness and toxic side effects still limit the prognosis of ACC [5]. Thus, it is very important to obtain reliable biomarkers that can predict the prognosis and drug efficacy of ACC.

In recent years, bioinformatic analyses and high-throughput microarray technology have been broadly applied to distinguish genetic changes at the genome level, which can overcome the sample size issue and assist us to seek reliable tumor biomarkers. However, at present, there is still lack of effective, reliable and clinically applicable biomarkers, which can well predict the prognosis and therapeutic effect of ACC. In the present study, we screened differentially expressed genes (DEGs) related to the prognosis of ACC by bioinformatics analysis from TCGA and GEO databases and constructed an effective prognostic prediction signature. Moreover, we further identified the pivotal gene in the prognostic signature and confirmed its potential value on predicting malignant progression, prognosis and mitotane efficacy in ACC.

## Materials and methods

### Data collection

RNA sequencing expression profiles of ACC patients were obtained from the TCGA database (https://portal.gdc.cancer.gov/, 92 ACC samples) and the GEO database (https://www.ncbi.nlm.nih.gov/geo/, 58 ACC samples and 5 normal samples). The concrete clinical information of TCGA ACC patients was presented in Additional file 2: Table S1.

Furthermore, the Affiliated Hospital of Qingdao University (AHQU)–ACC cohort including 29 ACC tissues and 10 normal adrenal tissues were obtained from the Department of Urology, AHQU, P. R. China (January 2000 to October 2018). Informed consent was acquired from all patients and the research protocol was approved by the Ethics Committee of AHQU (No. QYFY WZLL 26,516). All the patients were received retroperitoneal laparoscopic resection of adrenal tumor and did not receive chemotherapy or radiotherapy before surgery. The clinical information of AHQU–ACC cohort was presented in Table 1.

**Table 1:** GMNN expression correlates with clinicopathological characteristics of adrenocortical carcinoma (ACC)

Clinicopathological characteristics	Variable	Total (29)	GMNN expression		*P value*
			Low	High	
Age at diagnosis	< 60	12	2	10	
	≥ 60	17	8	9	***0.291***
Gender	Male	18	7	11	
	Female	11	3	8	***0.548***
Stage	I	4	3	1	
	II	2	1	1	
	III	5	3	2	
	IV	18	3	15	***0.011***
T	T1	4	3	1	
	T2	8	2	6	
	T3	10	3	7	
	T4	7	2	5	***0.327***
N	N0	18	8	10	
	N1	11	2	9	***0.164***
M	M0	12	7	5	
	M1	17	3	14	***0.028***
Ki-67	< 30	6	5	1	
	≥ 30	8	1	7	***0.014***

### Identification, validation and consensus clustering analysis of DEGs in ACC

The DEGs between ACC and normal samples in the GEO cohort were screened using GEO2R. LogFC (fold change) >|1| and adj. *P* values < 0.05 were considered to be statistically significant. Subsequently, we put overlapped DEGs into the STRING (Search Tool for the Retrieval of Interacting Gene) to formulate a PPI network [6]. Cytoscape (version 3.4.2) was used to draw a PPI network and identify the hub gene in the PPI network [7].

The expression levels of DEGs were further analyzed in TCGA cohort. To validate the correlation between the prognosis of ACC patients and the expression levels of DEGs, ACC patients were divided into various clusters by consensus clustering analysis with “ConsensusClusterPlus”, and the Kaplan–Meier algorithm was performed to explore the survival results among various clusters.

### Construction of a risk signature predicting prognosis for ACC patients

We performed the Univariate Cox regression analysis by using the R package “survival” and selection operator (LASSO) Cox regression to establish the optimal DEGs-based signature for predicting the prognosis in ACC patients with the R package “glmnet”. The DEGs-based signature risk score = Ʃ(βi × Expi) (*i* = the number of genes). Kaplan–Meier curves was used to evaluate the survival differences between the high and low risk groups. The survival ROC package was used to reveal the receiver operating characteristic curve (ROC) in order to evaluate the signature’s prediction accuracy. Univariate and multivariate Cox regression analyses were adopted to evaluate the prognostic significance of this signature.

Besides, we constructed a prognostic nomogram to evaluate the probability of 1-, 2-, and 3-, 5-year overall survival (OS) for ACC patients with the R package. Calibration curves were used to examine the concordance between the predicted and the actual survival rates.

### Validation of the hub gene associated with malignant progression and prognosis in ACC

The expression levels of hub gene–GMNN between normal samples and ACC samples in the TCGA ACC cohort were analyzed by Wilcoxon test. Kaplan–Meier curves and Cox regression analyses were also performed to evaluate the correlation between GMNN expression and clinicopathological features and prognosis in the TCGA ACC cohort.

Immunohistochemistry (IHC) was used to detect the expression levels of *GMNN* protein in the AHQU–ACC cohort. After being deparaffinized and boiled in sodium citrate buffer (0.01 mol/L, PH = 6.0) in the microwave for ten minutes as the use of antigen retrieval, tissue sections incubated with anti-*GMNN* antibody [ab104306] (Abcam, USA, 1:100) overnight at 4 °C. Biotinylated secondary antibody (goat–anti‐mouse–IgG, dilution 1:300) was then added for 30 min. Sections were stained by the avidin–biotin–peroxidase method and using 3–3’-diaminobenzidine as chromogen. Normal skin and lymph node tissues were used as positive controls for GMNN antibodies. Negative controls were performed using non-immunized mouse serum, omitting the primary antibodies.

Five fields of view were randomly selected by pathologist independently. The pathologist estimated the score according to the degree of staining (0: weakly, 1: moderately, 2: strongly) and the percentage of staining (0: 0–5%, 1: 5–50%, 2: 50–100%), and further calculated the mean score of the five fields respectively [8]. The final immunostaining score was the sum of staining intensity score and staining percentage score. The final immunostaining score > 2 was considered high expression of GMNN.

## Results

### Identification of DEGs and construction of DEGs-based prognostic signature for ACC

**Fig. 1:**
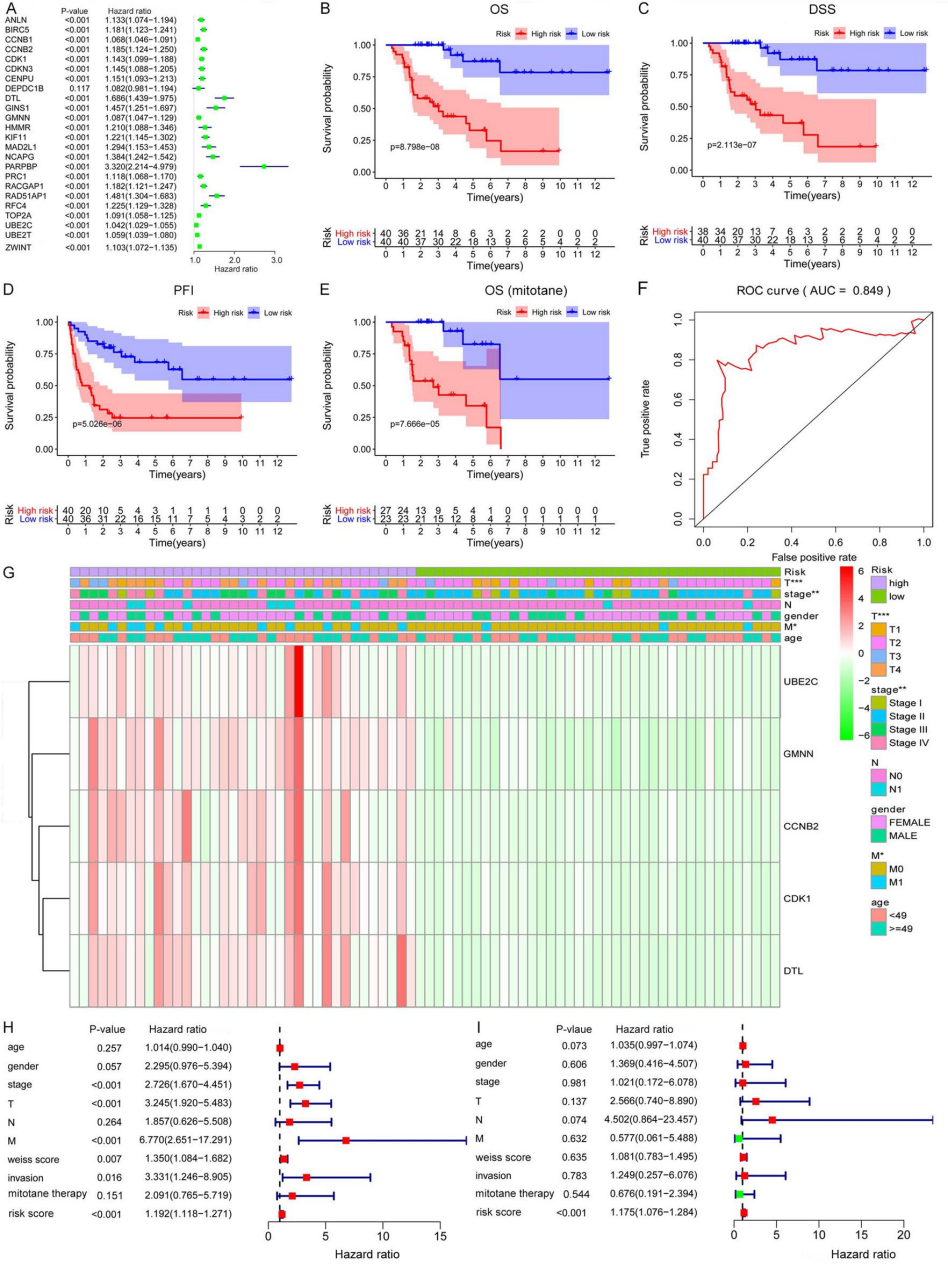
**Construction of a DEGs based signature to predict the prognosis of ACC**. (**A**) Univariate Cox regression analysis showed that 23 DEGs are closely associated with the OS of ACC patients. (**B–D**) Kaplan–Meier curves revealed that the high-risk group had significantly shorter OS, DSS, and PFI compared with the low-risk group. (**E**) In addition, high-risk scores also indicate poor OS in ACC patients who received mitotane therapy. (**F**) ROC curves showed that the AUC of the risk score was 0.849. (**G**) Heatmap showed that a significant difference was found between the high- and low-risk groups for the stage, T, and M. (**H**) Univariate Cox regression analysis showed that stage, T, M, Weiss score, invasion, and risk score were significantly related to the survival of ACC patients. (**I**) Multivariate Cox regression analysis demonstrated that the signature could serve as an independent prognostic predictor in ACC. *P < 0.05; **P < 0.01; ***P < 0.001; *DEG* differentially expressed gene, *ACC* adrenocortical carcinoma, *OS* overall survival, *DSS* disease-specific survival, *PFI* progression-free interval, *ROC* receiver operating characteristic curve, *AUC* area under curve

457 DEGs were identified in the GEO–ACC cohort including 377 downregulated genes and 80 upregulated genes. Then, a total of 28 DEGs were screened in the most significant module by using Cytoscape. Among these 28 DEGs, 24 genes were most differentially expressed (Additional file 1: Fig. S1) and related to the OS of ACC patients in TCGA ACC cohort (Fig. 1A). Multivariate Cox and lasso regression analysis were then used to construct a DEGs-based prognostic signature, which contained 5 genes including CCNB2 (cyclin B2), CDK1 (cyclin dependent kinase 1), DTL (denticleless E3 ubiquitin protein ligase homolog), GMNN (geminin DNA replication inhibitor), and UBE2C (ubiquitin conjugating enzyme E2C). The coefficient of prognostic risk score was as follows:(0.0294 × the expression level value of CCNB2) + (0.0369 × the expression level value of CDK1) + (0.0762 × the expression level value of DTL) + (0.843 × the expression level value of GMNN + (0.0099 × the expression level value of UBE2C).

Survival analyses indicated that high-risk scores were associated with poor OS, disease-specific survival (DSS), and progression-free interval (PFI) of ACC (Fig. 1B–D). Furthermore, the risk score was positively correlated with the treatment efficacy of mitotane for ACC patients (*P* = 7.666e-05, Fig. 1E). ROC curve analysis showed that the AUC was 0.849, which indicated excellent predictive accuracy of the prognostic signature (Fig. 1F). Moreover, the heatmap indicated that risk scores of the signature were closely related to tumor stage, T, and M in ACC patients (*P* < 0.05, Fig. 1G).

### The DEGs-based signature was an independent prognostic factor for ACC

Univariate and multivariate Cox regression analyses were used to assess the prognostic factors related to ACC including the DEGs-based signature and clinicopathological features. Univariate Cox regression showed that stage (*P* < 0.001), T (*P* < 0.001), M (P < 0.001), Weiss score (*P* < 0.05), invasion (*P* < 0.05), and risk scores (*P* < 0.001) were significantly related to the OS of ACC patients (Fig. 1H). Multivariate Cox regression analysis confirmed that the DEG-based signature was an independent prognostic factor for ACC patients (*P* < 0.001, Fig. 1I).

### High expression of GMNN was correlated with clinicopathologic outcomes and poor prognosis in ACC patients

**Fig. 2:**
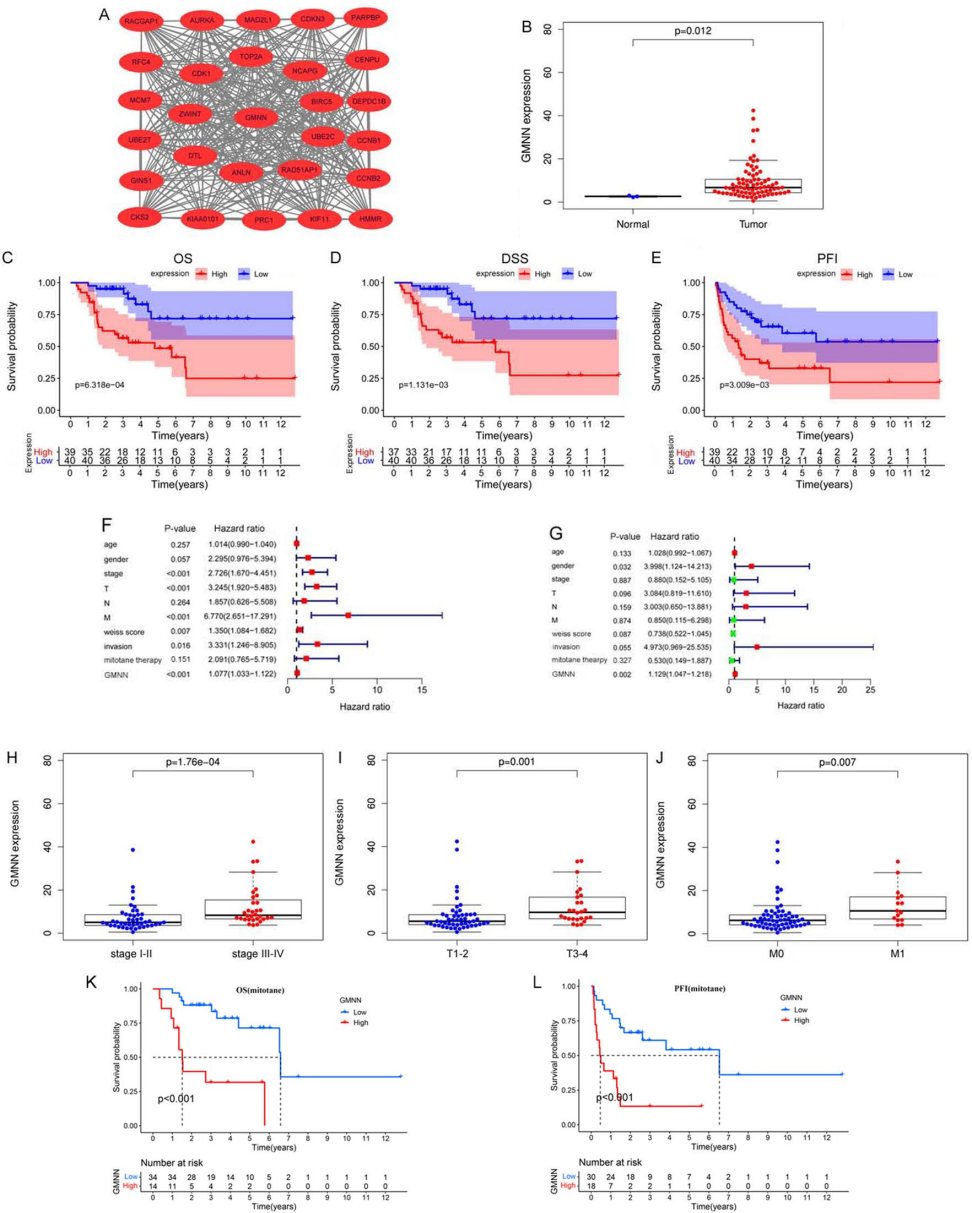
**Identification of the most significant module and validation of** ***GMNN*** **in the TCGA cohort**. (**A**) The most significant module and *GMNN* as a hub gene were recognized in the PPI network. **B** *GMNN* was differentially expressed. (**C–E**) GMNN was significantly related to poor OS, DSS, and PFI in TCGA ACC sample. (**F**, **G**) Univariate Cox regression and multivariate Cox regression analyses indicated that *GMNN* was independently related to OS of TCGA ACC patients. (**H–J**) The expression level of *GMNN* was associated with stage, T, and M in ACC. (**K**, **L**) GMNN was closely related to OS and PFI of ACC patients treated with mitotane. *OS* overall survival, *DSS* disease-specific survival, *PFI* progression-free interval, *ACC* adrenocortical carcinoma

MCODE analysis indicated that GMNN was a hub gene in the most significant module (Fig. 2A). TCGA–ACC cohort showed that there was a significant difference in the expression of GMNN between ACC samples and normal samples and GMNN was significantly related to OS (*P* = 6.318e-04), DSS (*P* = 1.131e-03), and PFI (*P* = 3.009e-03) for ACC patients (Fig. 2B–E). GMNN was also correlated with the stage, T and M in ACC and univariate and multivariate Cox regression showed that the GMNN was an independent prognostic factor for ACC patients (*P* < 0.05, Fig. 2F–J). More importantly, GMNN was closely related to the OS (*P* < 0.001) and PFI (*P* < 0.001) of ACC patients treated with mitotane (Fig. 2K, L).

**Fig. 3:**
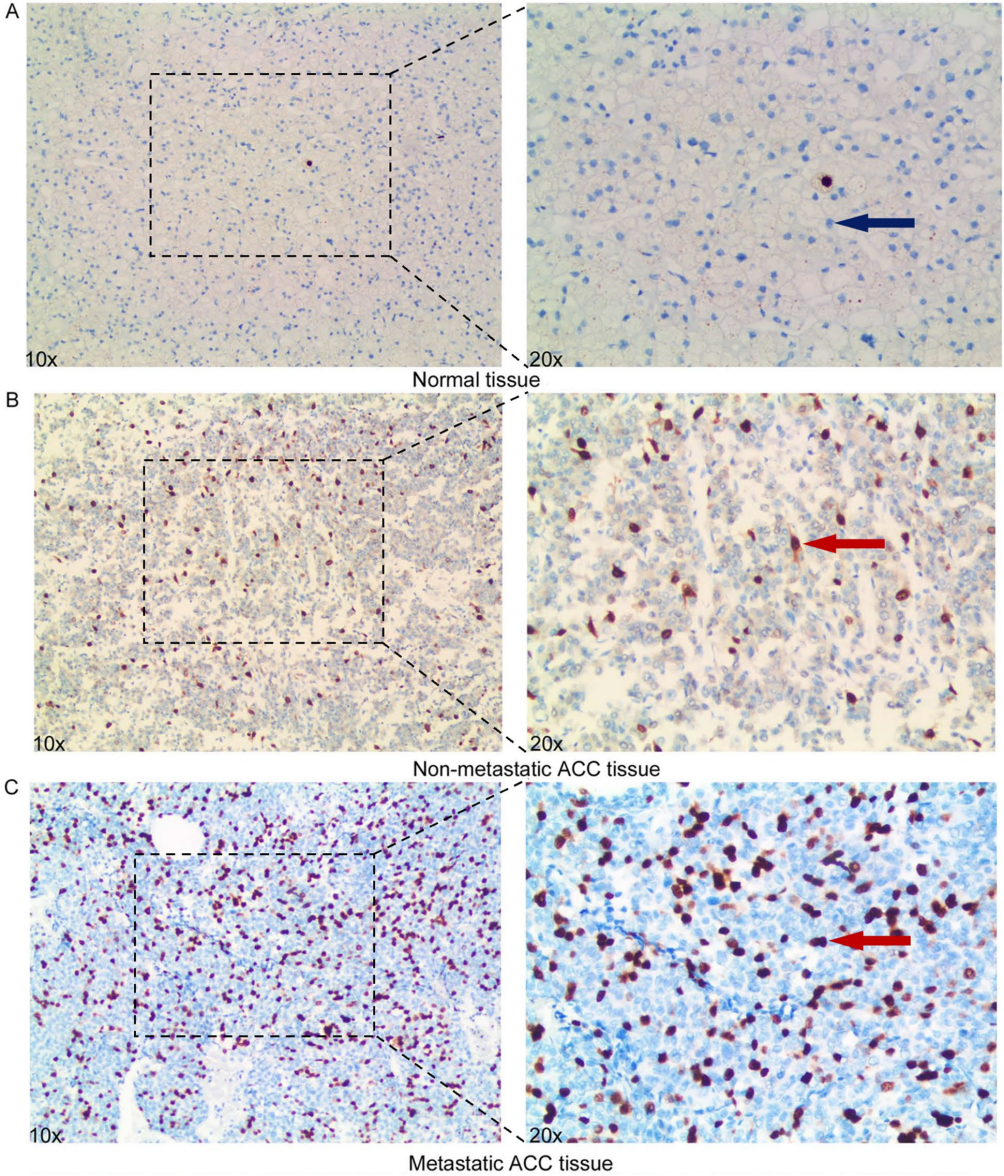
**The protein expression levels of** ***GMNN*** **in our ACC and normal tissues**. *GMNN* was negative or weakly expressed in normal tissues (**A**), moderately expressed in non-metastatic ACC tissues (**B**), and highly expressed in metastatic ACC tissues (**C**). Red arrow: positively stained ACC cells; blue arrow: negatively stained ACC cells. *ACC* adrenocortical carcinoma

We further validated the protein expressions of GMNN in 29 ACC samples and 10 normal adrenal samples from AHQU ACC cohort by IHC. We observed that GMNN was aberrantly expressed in 65.52% of ACC patients and the protein expression level of GMNN was significantly higher in ACC tissues than that in normal adrenal tissues (*P* < 0.05). In addition, in ACC cells, the immunoexpressions of GMNN were primarily in the nucleus (Fig. 3A–C). To evaluate the role of GMNN expression in ACC, the correlations between GMNN immunoreactivity and clinicopathologic features were analyzed and summarized in Table 1. Interestingly, we found that GMNN expression was significantly correlated with stage (*P* = 0.011), M (*P* = 0.028) and Ki67 index (*P* = 0.014), indicating that GMNN may play an important role in the malignant progression and metastasis of ACC.

### Construction of a nomogram predicting prognosis for ACC

**Fig. 4:**
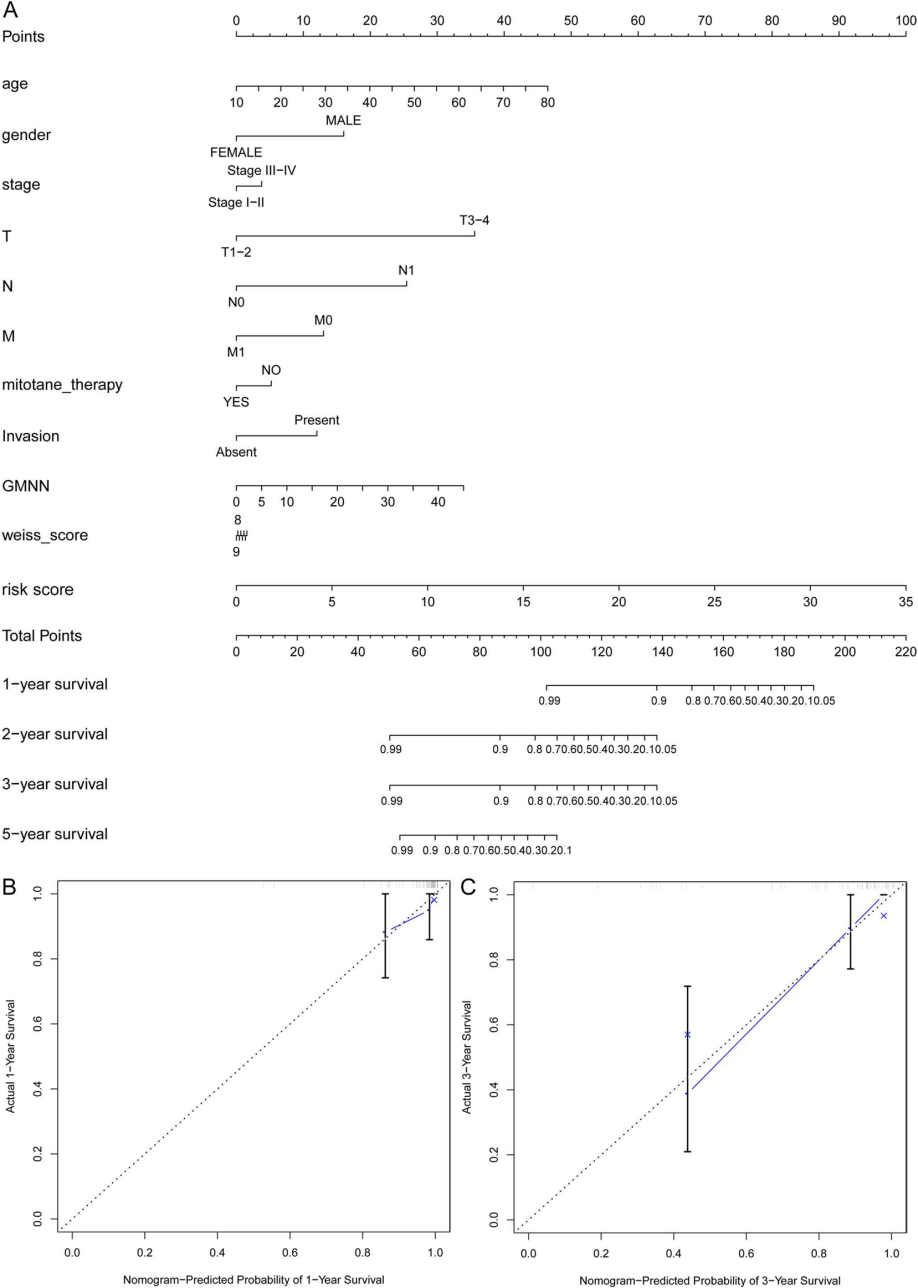
**A prognostic nomogram with clinical features,** ***GMNN*** **expression levels, and DEGs-based signature risk score was established for ACC**. (**A**) The nomogram could superiorly predict 1-, 2-, and 3-, 5-year OS of ACC patients. (**B–C**) The calibration plots also demonstrate excellent agreement between prediction and observation for the 1- and 3-year OS probabilities of the TCGA ACC patients. *DEG* differentially expressed gene, *ACC* adrenocortical carcinoma, *OS* overall survival

To obtain a clinically applicable tool for monitoring the prognosis of ACC patients, we also constructed a nomogram containing the DEGs-based signature, GMNN expression levels and clinicopathologic features (including age, stage, gender, TNM, invasion, mitotane treatment, and Weiss score). Our results showed that the nomogram could well predict the 1-, 2-, 3-, 5-year OS for ACC patients (Fig. 4A). The calibration plots also demonstrated excellent coincidence between observation and prediction for the 1- and 3-year OS probabilities for ACC patients (Fig. 4B, C).

## Discussion

ACC is characterized by low incidence rate, aggressive behaviors and poor prognosis. Thus, it is urgent to explore novel and reliable biomarkers which can well predict prognosis and provide potential therapeutic target for ACC [9]. Previous studies have already identified a number of potential hub genes in ACC by using bioinformatic methods. Guo et al. described a prognostic signature for ACC containing 9 genes, including CCNB1 (cyclin B1), CDK1, TOP2A (topoisomerase IIα), CCNA2 (cyclin A2), CDKN3 (cyclin‐dependent kinase inhibitor 3), MAD2L1 (mitosis arrest‐deficient 2-like 1), RACGAP1 (rac GTPase activating protein 1), BUB1 (benzimidazole 1 homolog beta) and CCNB2 [10]. Xu et al. suggested that ZWINT (ZW10 interactor), PRC1 (protein regulator of cytokinesis 1), CDKN3 (cyclin-dependent kinase inhibitor 3), CDK1 and CCNA2 were involved in early recurrence of ACC [2]. In our study, we identified 24 DEGs correlated with prognosis of ACC and constructed a five gene (CCNB2, CDK1, DTL, GMNN, and UBE2C)-based prognostic signature. Different from previous studies, for the first time we found that GMNN was the core gene in this signature, which was closely correlated with clinicopathologic outcomes and prognosis of ACC.

GMNN is an important gene for cell cycle regulation. GMNN encodes a protein inhibits histone acetyltransferase activity of KAT7/HBO1 in a CDT1-dependent manner, inhibiting DNA replication licensing and histone H4 acetylation, enrolling them in cell proliferative control. Previous studies have demonstrated that GMNN expression was correlated with prognosis in oral squamous cell carcinoma, squamous cell carcinoma of the tongue, and melanoma [11–13]. Blanchard et al. found that upregulated GMNN acted as an oncogene that promotes cytokinesis failure and the progression of breast tumors and thus became an effective therapeutic target for aggressive breast cancer [14]. However, relevant research on GMNN in ACC is still limited. In the present study, we found that GMNN was significantly overexpressed in ACC tissues and was closely related to the pathological features and prognosis. Previous studies demonstrated that high GMNN/Ki-67 ratio indicated a short G1 phase and a high rate of cell proliferation [15–18]. Interestingly, we found that GMNN expression was significantly correlated with Ki-67 index in ACC. Furthermore, inhibiting GMNN activity could selectively arrest cancer cell proliferation by inducing DNA damage-mediated apoptosis and DNA re-replication without affecting the normal cells [19]. Thus, we speculate that GMNN may promote the malignant progression of ACC by regulating the cell cycle and promoting cell proliferation. However, the underlying mechanism of GMNN affecting the prognosis of ACC still needs to be further studied.

Mitotane is the only available drug approved for the treatment of advanced ACC. Considering the toxicity and side effects, it is very important to screen ACC patients who can benefit from mitotane [20, 21]. However, there is still a lack of biomarkers for predicting the efficacy of mitotane in ACC. Another interesting finding in our study was that GMNN showed close relation with the OS and PFI of ACC patients treated with mitotane. Moreover, a nomogram containing GMNN expression levels and mitotane treatment was constructed and showed a relatively high accuracy of prediction. These findings indicated that GMNN could be used as a potential biomarker for predicting mitotane efficacy in ACC.

There are still some limitations in this study. First, as a retrospective study, it is inevitable to have possible selection bias and low statistical ability; secondly, as this study mainly aimed to explore the potential clinical values of selected hub genes in the diagnosis and therapy of ACC, the details of their mechanisms were not comprehensively explored, especially GMNN with very limited number of previous studies of its effect on ACC. In addition, bioinformatics research based on public database needs further experimental research to explore its potential role and deep-seated molecular mechanism.

In conclusion, we constructed a five DEGs (CCNB2, CDK1, DTL, GMNN, and UBE2C)-based prognostic signature for ACC and first identified GMNN as a novel tumor biomarker for predicting the malignant progression, mitotane efficacy, and prognosis of ACC.

## Supplementary Information

**Additional file 1.** Differential clinical outcomes of ACC patients in the two different clusters. Heatmap was generated to validate the expression levels of DEGs between ACC and normal samples in the TCGA cohort. *ACC* adrenocortical carcinoma.

**Additional file 2: Table S1.** TCGA–adrenocortical carcinoma (ACC) patient characteristics.

## Data Availability

The data used to support the findings of this study is included within the article, and the data are available from the corresponding author upon request.

## Abbreviations

ACC: Adrenocortical carcinoma
IHC: Immunohistochemical
GMNN: Geminin DNA replication inhibitor
DEGs: Differentially expressed genes
GEO: Gene expression omnibus
TCGA: The cancer genome atlas
PPI: Protein–protein interactions
OS: Overall survival
DSS: Disease-specific survival
PFI: Progression-free interval
CCNB2: Cyclin B2
CDK1: Cyclin dependent kinase 1
DTL: Denticleless E3 ubiquitin protein ligase homolog
UBE2C: Ubiquitin conjugating enzyme E2 C
AUC: Area under the curve
STRING: Search tool for the retrieval of interacting gene
K–M: Kaplan–Meier
ENSAT: European network for the study of adrenal tumors
LASSO: Least absolute shrinkage and selection operator
ROC: Receiver operating characteristic curve
CCNB1: Cyclin B1
TOP2A: Topoisomerase IIα
CCNA2: Cyclin A2
CDKN3: Cyclin‐dependent kinase inhibitor 3
MAD2L1: Mitosis arrest‐deficient 2-like 1
RACGAP1: Rac GTPase activating protein 1
BUB1: Benzimidazole 1 homolog beta
ZWINT: ZW10 interactor
PRC1: Protein regulator of cytokinesis 1
CDKN3: Cyclin-dependent kinase inhibitor 3

## References

[1] Calissendorff J, Calissendorff F, Falhammar H (2016). Adrenocortical cancer: mortality, hormone secretion, proliferation and urine steroids—experience from a single centre spanning three decades. BMC Endocr Disord.

[2] Xu WH, Wu J, Wang J, Wan FN, Wang HK, Cao DL (2019). Screening and identification of potential prognostic biomarkers in adrenocortical carcinoma. Front Genet.

[3] Fassnacht M, Johanssen S, Quinkler M, Bucsky P, Willenberg HS, Beuschlein F (2009). Limited prognostic value of the 2004 international union against cancer staging classification for adrenocortical carcinoma: proposal for a revised TNM classification. Cancer.

[4] Schulick RD, Brennan MF (1999). Long-term survival after complete resection and repeat resection in patients with adrenocortical carcinoma. Ann Surg Oncol.

[5] Bedrose S, Daher M, Altameemi L, Habra MA (2020). Adjuvant therapy in adrenocortical carcinoma: reflections and future directions. Cancers.

[6] Szklarczyk D, Morris JH, Cook H, Kuhn M, Wyder S, Simonovic M (2017). The STRING database in 2017: quality-controlled protein-protein association networks, made broadly accessible. Nucleic Acids Res.

[7] Smoot ME, Ono K, Ruscheinski J, Wang PL, Ideker T (2011). Cytoscape 28: new features for data integration and network visualization. Bioinformatics.

[8] Taheri D, Zahavi DJ, Del Carmen RM, Meliti A, Rezaee N, Yonescu R (2016). For staining of ALK protein, the novel D5F3 antibody demonstrates superior overall performance in terms of intensity and extent of staining in comparison to the currently used ALK1 antibody. Virchows Arch.

[9] Assié G, Letouzé E, Fassnacht M, Jouinot A, Luscap W, Barreau O (2014). Integrated genomic characterization of adrenocortical carcinoma. Nat Genet.

[10] Guo J, Gu Y, Ma X, Zhang L, Li H, Yan Z (2020). Identification of hub genes and pathways in adrenocortical carcinoma by integrated bioinformatic analysis. J Cell Mol Med.

[11] Jiang Q, Yu YC, Ding XJ, Luo Y, Ruan H (2014). Bioinformatics analysis reveals significant genes and pathways to target for oral squamous cell carcinoma. Asian Pacific J Cancer Prev.

[12] Quan L, Shi J, Tian Y, Zhang Q, Zhang Y, Zhang Y (2015). Identification of potential therapeutic targets for melanoma using gene expression analysis. Neoplasma.

[13] Zhou RS, Zhang EX, Sun QF, Ye ZJ, Liu JW, Zhou DH (2019). Integrated analysis of lncRNA-miRNA-mRNA ceRNA network in squamous cell carcinoma of tongue. BMC Cancer.

[14] Blanchard Z, Malik R, Mullins N, Maric C, Luk H, Horio D (2011). Geminin overexpression induces mammary tumors via suppressing cytokinesis. Oncotarget.

[15] Cates JM, Memoli VA, Gonzalez RS (2015). Cell cycle and apoptosis regulatory proteins, proliferative markers, cell signaling molecules, CD209, and decorin immunoreactivity in low-grade myxofibrosarcoma and myxoma. Virchows Arch.

[16] de Andrade BA, León JE, Carlos R, Delgado-Azañero W, Mosqueda-Taylor A, de Almeida OP (2013). Expression of minichromosome maintenance 2, Ki-67, and geminin in oral nevi and melanoma. Ann Diagn Pathol.

[17] Kushwaha PP, Rapalli KC, Kumar S (2016). Geminin a multi task protein involved in cancer pathophysiology and developmental process: a review. Biochimie.

[18] Wharton SB, Hibberd S, Eward KL, Crimmins D, Jellinek DA, Levy D (2004). DNA replication licensing and cell cycle kinetics of oligodendroglial tumours. Br J Cancer.

[19] Zhu W, Depamphilis ML (2009). Selective killing of cancer cells by suppression of geminin activity. Can Res.

[20] Shariq OA, McKenzie TJ (2021). Adrenocortical carcinoma: current state of the art, ongoing controversies, and future directions in diagnosis and treatment. Ther Adv Chronic Dis.

[21] Terzolo M, Fassnacht M (2022). Endocrine tumours: our experience with the management of patients with non-metastatic adrenocortical carcinoma. Eur J Endocrinol.

