# Supplementary figures and images for: High expression of GMNN predicts malignant progression and poor prognosis in ACC
Source: Eur J Med Res. 2022 Dec 20;27:301. doi: 10.1186/s40001-022-00950-2 (PMC9764478; doi:10.1186/s40001-022-00950-2)

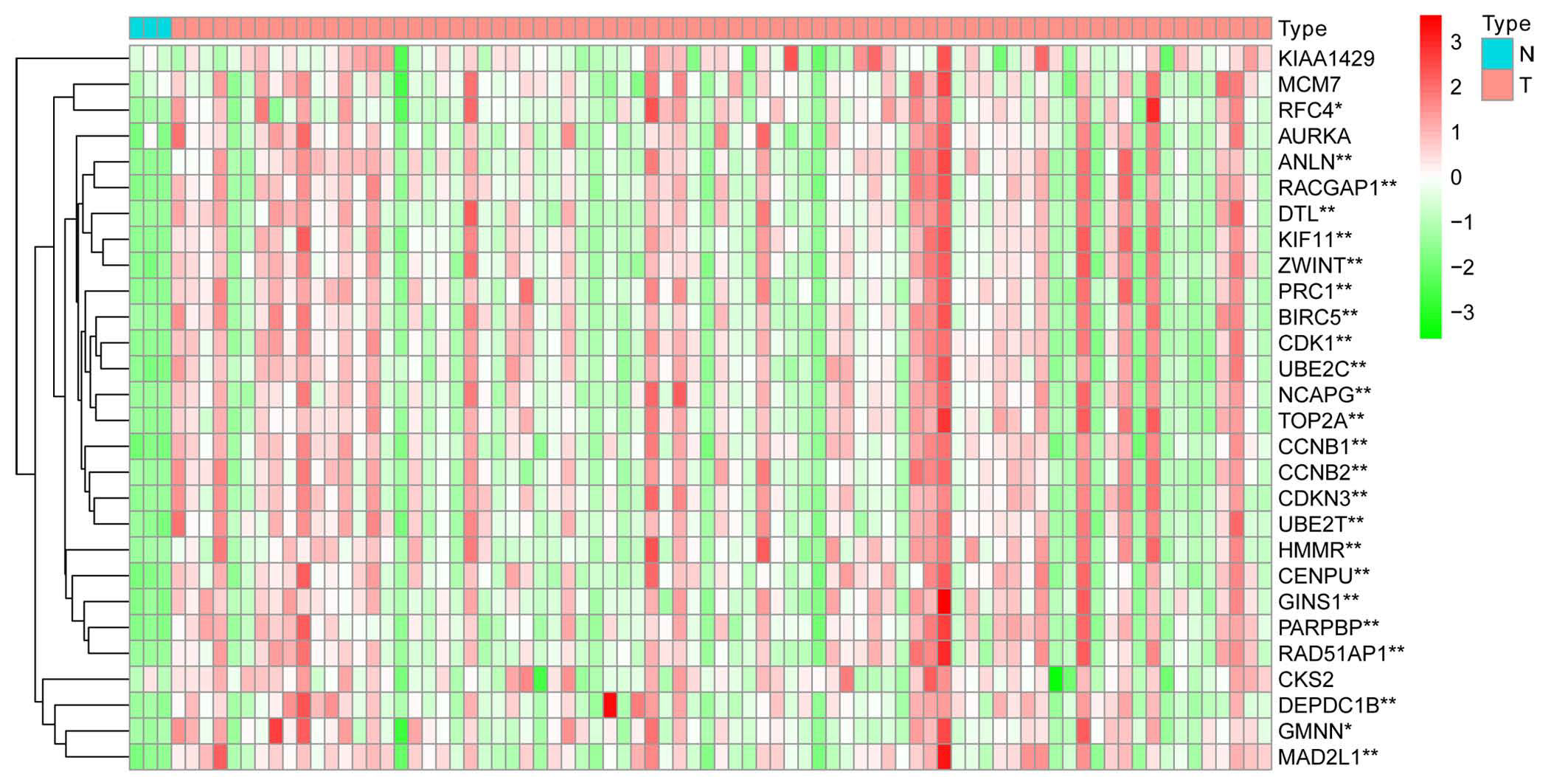

Supplement: Supplementary file 1 — Additional file 1. Differential clinical outcomes of ACC patients in the two different clusters. Heatmap was generated to validate the expression levels of DEGs between ACC and normal samples in the TCGA cohort. ACC adrenocortical carcinoma. [file 40001_2022_950_MOESM1_ESM.tif]
