# Supplementary material for: High expression of GMNN predicts malignant progression and poor prognosis in ACC
Source: Eur J Med Res. 2022 Dec 20;27:301. doi: 10.1186/s40001-022-00950-2 (PMC9764478; doi:10.1186/s40001-022-00950-2)
Supplement: Supplementary file 2 — Additional file 2: Table S1. TCGA–adrenocortical carcinoma (ACC) patient characteristics. [file 40001_2022_950_MOESM2_ESM.docx]

**Table S1 TCGA-adrenocortical carcinoma(ACC) patient characteristics.**

| Clinical characteristics |  | Total (92) | % |
| --- | --- | --- | --- |
| Age |  | 49 (14-83) |  |
| Gender | Female | 60 | 65.22 |
|  | Male | 32 | 34.78 |
| Grade | Unknown |  |  |
| Stage | I | 9 | 9.78 |
|  | II | 44 | 47.83 |
|  | III | 19 | 20.65 |
|  | IV | 18 | 19.57 |
|  | Unknown | 2 | 2.17 |
| T | T1 | 9 | 9.78 |
|  | T2 | 49 | 53.26 |
|  | T3 | 11 | 11.96 |
|  | T4 | 21 | 22.83 |
|  | Unknown | 2 | 2.17 |
| N | N0 | 80 | 86.96 |
|  | N1 | 10 | 10.87 |
|  | Unknown | 2 | 2.17 |
| M | M0 | 72 | 78.26 |
|  | M1 | 18 | 19.57 |
|  | Unknown | 2 | 2.17 |
| Invasion of tumor capsule | Absent | 35 | 38.05 |
|  | Present | 48 | 52.17 |
|  | Unknown | 9 | 9.78 |
| Mitotane therapy | No | 35 | 38.04 |
|  | Yes | 55 | 59.79 |
|  | Unknown | 2 | 2.17 |
| Radiation therapy | No | 71 | 77.17 |
|  | Yes | 18 | 19.57 |
|  | Unknown | 3 | 3.26 |
| Weiss score | 2 | 3 |  |
|  | 3 | 12 |  |
|  | 4 | 11 |  |
|  | 5 | 8 |  |
|  | 6 | 11 |  |
|  | 7 | 10 |  |
|  | 8 | 9 |  |
|  | 9 | 5 |  |
|  | Unknown | 23 |  |
